# Using Network Science to Understand the Aging Lexicon: Linking Individuals' Experience, Semantic Networks, and Cognitive Performance

**DOI:** 10.1111/tops.12586

**Published:** 2022-01-18

**Authors:** Dirk U. Wulff, Simon De Deyne, Samuel Aeschbach, Rui Mata

**Affiliations:** ^1^ Faculty of Psychology University of Basel; ^2^ Center for Adaptive Rationality Max Planck Institute for Human Development; ^3^ Melbourne School of Psychological Sciences University of Melbourne

**Keywords:** Semantic networks, Cognitive aging, Individual differences

## Abstract

People undergo many idiosyncratic experiences throughout their lives that may contribute to individual differences in the size and structure of their knowledge representations. Ultimately, these can have important implications for individuals' cognitive performance. We review evidence that suggests a relationship between individual experiences, the size and structure of semantic representations, as well as individual and age differences in cognitive performance. We conclude that the extent to which experience‐dependent changes in semantic representations contribute to individual differences in cognitive aging remains unclear. To help fill this gap, we outline an empirical agenda that utilizes network analysis and involves the concurrent assessment of large‐scale semantic networks and cognitive performance in younger and older adults. We present preliminary data to establish the feasibility and limitations of such empirical, network‐analytical approaches.

## Abstract 2

Whether it is possible to define a rational standard in decision making and, if yes, whether such a standard is achievable by finite agents (such as humans) has been a hotly debated issue. This special issue offers an overview of some promising modern approaches to these questions, taking advantage of the latest developments in decision theory.

We review evidence that suggests links between individual experiences, semantic representations, and age differences in cognitive performance, and present an empirical agenda and pilot data involving the assessment of large‐scale, individual semantic networks.

## 1 Introduction

From childhood and adolescence onwards, the average human reads a couple of books each year, watches hundreds of hours of TV, and spends many hours on social media (Twenge, Martin, & Spitzberg, 2019), leading to the accumulation of a large and, potentially, largely unique, set of experiences during a lifetime. To what extent does the accumulation of such idiosyncratic experiences contribute to individual differences in thought and judgment across the life span?

Aging research has long realized not only the importance of describing the modal changes in cognition across the life span but also that “cognitive development in adulthood and old age differs substantially from person to person and is malleable within individuals” (Lindenberger, 2014 p. 576). Despite the field's direct acknowledgment of interindividual differences, we still know little about the sources of such differences and the extent to which idiosyncratic life experiences contribute to cognitive performance. Crucially, some voices have raised the possibility that cumulative experience and the cognitive representations they give rise to are a major factor underlying typical age‐related patterns, such as decreased memory performance with increased age (Buchler & Reder, 2007; Ramscar, Hendrix, Shaoul, Milin, & Baayen, 2014).

In this article, we propose that network science can be instrumental in illuminating the effects of cumulative experience on individual differences in cognition. To this end, we first review the existing literature on the links between cumulative experience, cognitive representation, and cognitive performance. We then identify the lack of direct assessment of individuals' mental representations as a central limitation, and propose an empirical agenda that utilizes network analysis to fill this gap. Next, we present a proof‐of‐concept study that illustrates the feasibility and limitations of our approach involving the elicitation of large‐scale semantic networks from single individuals. Finally, we discuss the challenges and implications of the outlined research agenda to understand individual and age differences.

## 2 From experience to cognitive performance: An overview of the current literature

**Fig. 1:**
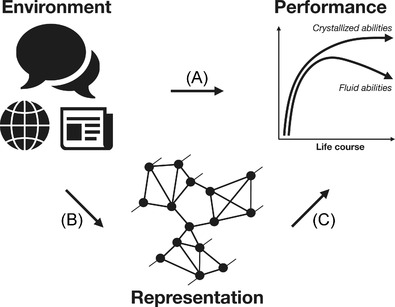
Research pathways that have linked environment, representation, and cognitive performance across the life span. Pathway (A) represents research studying the association between the environment and cognitive performance without explicit consideration of the underlying mental representations. Pathway (B) represents research on the association between environmental exposure and mental representations. Pathway (C) represents research on the association between mental representations and cognitive performance. We propose that pathways (B) and (C) can particularly benefit from a network analytic approach.

In this section, we assess the current state of the psychological literature that has investigated the links between environmental exposure, the size and structure of semantic representations, and cognitive performance across the life span (see Fig. 1). Our goal is both to provide a brief overview of the current literature and to highlight the main gaps that must be filled to understand the role of individual experience in cognitive representations and performance.

There are several lines of research supporting a close link between cumulative experience and cognitive performance (see Fig. 1, pathway A: Environment–Performance), of which three stand out. First, research that summarizes the association between print exposure and reading performance in children and young adults underlines the tremendous impact of experience on linguistic proficiency. Specifically, meta‐analytic results show moderate to strong correlations between print exposure and reading comprehension or spelling (Mol & Bus, 2011). This work supports the idea of an “upward spiral,” such that individuals who are more exposed to written text become more proficient in reading and comprehension, which, in turn, leads to increased print exposure, further increasing linguistic proficiency. Second, one of the best documented findings in aging research concerns the observed increases in crystallized abilities across the life span, as reflected in vocabulary size (Verhaeghen, 2003, for a meta‐analysis). This work suggests that even as adults, individuals continue to expand their vocabulary store throughout their lives (Brysbaert, Stevens, Mandera, & Keuleers, 2016). This is compatible with the idea that such vocabulary growth is related to individuals' lifetime formal or informal education. Notably, such increases in knowledge seem to have general implications for cognitive performance. For example, older adults are more likely to rely on prediction in reading because of their additional reading experience (Huettig & Pickering, 2019; but see Wlotko, Federmeier, & Kutas, 2012). Third, research on expertise suggests that increased cumulative experience leads to domain‐specific memory performance (Sala & Gobet, 2017, for a meta‐analysis), such as experienced chess players being able to memorize both realistic and random chess positions better than novices (Gobet & Simon, 1996). All in all, such findings coalesce to make a strong case for the importance of environmental effects on cognitive performance and suggest that these effects are cumulative and, likely, domain specific.

Most, if not all, psychologists will find it trivial to state that the effects of cumulative experience are somehow mediated by its effects on mental representations and processes (Fig. 1, pathway B, Environment–Representation). Despite the truism and the increasing consensus that the lexical‐semantic space continues to be shaped by personal linguistic experience throughout the life span (Rodd, 2020), only recently have researchers started to probe more deeply into the effects of cumulative experience on the structure of knowledge representations. Some researchers have adopted graph‐based approaches to capture potential structural changes to the mental lexicon that occur across the life span as a function of experience (for an overview, see Wulff, De Deyne, Jones, Mata, & The Aging Lexicon Consortium, 2019). The current literature suggests that cumulative experience has implications for not only the size but also the structure of mental representations (e.g., Cosgrove, Kenett, Beaty, & Diaz, 2021; Dubossarsky, De Deyne, & Hills, 2017). For example, Dubossarsky and colleagues conducted a network analysis of free association data from thousands of individuals (10 to 84 years of age), and found older adults' semantic networks were less connected (i.e., the words in the network have lower average degrees), less organized (i.e., the words in the network have a lower average local clustering coefficient), and less efficient (i.e., the shortest path length between any two words in the network is greater) relative to those of younger adults. Crucially, new methods are becoming available that promise ever greater insight and precision in mapping individual and group differences in the structure of mental representations (e.g., Benedek et al., 2017; Morais, Olsson, & Schooler, 2013; Zemla & Austerweil, 2018). Table 1 presents an overview of a few approaches to measuring individual‐level mental representations and we discuss in more detail pros and cons of each approach in the next section (cf. Section 3).

Finally, research is accumulating that establishes direct links between the structure of semantic representations and cognitive performance (Fig. 1, pathway C, Representation–Performance). For example, various studies on memory recall show impaired performance when words represented as nodes within a semantic network have lower clustering (Nelson, Bennett, Gee, Schreiber, & McKinney, 1993). More recent work has expanded this line of research to understand how lexical and semantic structure is crucial to individual cognitive performance in various domains, such as intelligence and creativity (He et al., 2021; Kenett & Faust, 2019). These more recent studies are particularly relevant because they have started relying on individual or small group estimates of mental representations and how macroproperties of representational networks impact cognitive performance. However, one should note that this work has not established a direct link between explicit measures of an individual's cumulative experience, such as books read or movies watched, and the size or structure of the mental representations—instead studies have typically relied on proxies for experience, such as age (Wulff et al., 2019).

All in all, these different lines of research support the idea that cumulative personal experience plays a crucial role in determining individual differences in cognitive performance across the life span. However, much of this work is correlational (Nation, 2017, for a similar critique) and not carried out at the level of the individual. As a result, the mechanisms that tie cumulative exposure to performance are still under investigation. We propose that to truly understand the consequences of individual experience on mental representation and cognitive performance, the concurrent assessment of individuals' unique environments, as well as their mental representations and cognitive performance, is needed. In this endeavor, network science provides a key tool to capturing individuals' mental representations, in particular, in the quantification of the connection patterns between mental concepts that can be empirically related to both experience and cognitive performance. In what follows, we offer an empirical agenda that uses network science tools to better understand the aging lexicon.

**Table 1:** Approaches to measuring individual‐level semantic networks

Paradigm	Description	Scope	Comparability	References
Verbal fluency	Individuals generate as many items from a given category as they can in a fixed period of time	Limited	High	(Zemla & Austerweil, 2018)
Relatedness judgments	Individuals rate the relation, i.e., similarity, between pairs of items	Limited	High	(Benedek et al., 2017; Roads & Love, 2020)
Free association (snowball)	Individuals generate one or more associations to word cues, which are participant generated	Broad	Low	(Morais et al., 2013)
Free association (fixed list)	Individuals generate one or more associations to experimenter‐generated word cues	Broad	High	See below

*Note*. *Scope* = Ability of the paradigm to provide coverage of a large set of semantic categories; *Comparability* = Ability of the paradigm to provide comparable coverage of semantic representation from different individuals.

## 3 An empirical agenda

As outlined above, the current literature has made large strides toward understanding the components linking cumulative experience to cognitive performance: We know that younger and older adults differ in the amounts and kinds of experiences, the contents and structure of mental representations, and that there are systematic age and individual differences in both fluid and crystallized performance. However, presently, we cannot confidently estimate the portion of individual differences in performance across the life span due to the accumulation of specific types of experience. What is missing, in our mind, is a concerted empirical agenda that investigates the Environment–Representation and the Representation–Performance pathways at the level of the individual, thus allowing us to quantify how a certain type or quantity of experience translates into the size or structure of cognitive representations, and, ultimately, into differences in cognitive performance. This goal presents challenges on several levels, and we discuss two major ones below: first, the challenge of measuring individuals' environments, and, second, the challenge of capturing the content and structure of single individuals' mental representations.

Concerning the challenge of measuring individuals' environments, one main limiting factor is currently the lack of available data. Put simply, the field lacks context‐aware longitudinal projects that provide a characterization of the environments experienced by individuals over time. Although unprecedented, large amounts of contextualized text and speech data are now available to scientists (e.g., Love, Dembry, Hardie, Brezina, & McEnery, 2017; Schröter & Schroeder, 2017), few of these data sets present data on the level of the individual or distinguish between age groups. Crucially, any individual's environment consists of not only linguistic information, but also rich multimodal sensorial information (e.g., De Deyne, Navarro, Collell, & Perfors, 2021). Thus, a major challenge for future research is to create individual‐annotated, multimodal corpora accessible for research. There is optimism in the field that the rise of the quantified‐self movement and the availability of new “digital tracing” methods can provide multiple data streams to feed computational modeling efforts that use these data to create models of individual's mental representations (Wulff et al., 2019). These models would be crucial to generate expectations about individuals' actual mental representations.

Concerning the challenge of mapping individuals' mental representations, and as discussed above, there are now different approaches to obtaining semantic networks in order to characterize the size and structure of mental representations (see Table 1). However, not all of these approaches are equally suited to accurately uncovering the full breadth and depth of a single individual's mental representation (scope) while ensuring comparability between individuals in the lexical‐semantic space covered (comparability). For example, approaches to derive networks derived from verbal fluency tasks, for example, using the U‐INVITE algorithm (Zemla & Austerweil, 2018), are limited to specific categories (e.g., concrete categories such as animals) and semantic relationships (e.g. is‐a). which may not be representative of the entire semantic network (De Deyne & Storms, 2008). In addition, learning about within‐category structure is not very informative about the links between categories. In turn, other approaches that derive networks from relatedness judgments require no intricate modeling and can be powerful in providing comparability between individuals because participants can be presented with the same pairs of concepts (Wulff, Hills, & Mata, 2018). However, asking individuals to provide ratings to all possible pairs becomes prohibitively demanding with even small sets of stimuli because this implies thousands of paired comparisons.^1^ Compared to other methods, free associations are a relatively economical basis for semantic networks and can provide broad scope. However, free‐association methods that ask individuals to generate associations in a snowball method (Morais et al., 2013) may reduce comparability across participants' networks due to path dependency in any individual's search of the representational store. In comparison, to generate networks from free associations with fixed, experimenter‐generated lists may provide a better choice in both scope and comparability. One should note that free association has also been the method of choice for most previous large‐scale assessments of aggregate semantic networks (De Deyne, Navarro, Perfors, Brysbaert, & Storms, 2019; Steyvers, Shiffrin, & Nelson, 2005).

All in all, our review of the literature, as well as assessment of the challenges above, highlights several future directions: first, providing a better description of the idiosyncratic experiences of individuals that can inform computational and learning models of linguistic and semantic cognition, and second, mapping the mental representations of single individuals that can later be matched to expectations about the role of experience in cognitive performance and the potential consequences. In what follows, we provide our own attempt at using network science to tackle the second challenge of mapping individual semantic networks and linking them to cognitive performance.

## 4 Estimating the Representation–Performance pathway: The MySWOW project

Our team is currently pursuing the *My Small World of Words* (MySWOW) project that aims to map the semantic networks of single individuals so as to be able to link the structural characteristics of each individual's mental representation and several aspects of cumulative experience and performance.

In what follows, we describe a small study that showcases and assesses the feasibility of such an approach. Our project and the procedure of our feasibility study was inspired by ongoing efforts to obtain word association norms for several languages in a large online citizen‐science project, the Small World of Words (SWOW) study (https://smallworldofwords.org). SWOW has already offered a set of useful linguistic resources for both Dutch (De Deyne, Navarro, & Storms, 2013) and English (De Deyne et al., 2019) and aims to contribute additional resources concerning 15 other languages, including German (https://smallworldofwords.org/de), in the future. The adoption of the same procedure for the large, population‐based SWOW and the study of individuals, MySWOW, promises future assessments of the comparability between the results from aggregate networks and those of single individuals or small groups.

We largely adopted the procedure of SWOW, which asks participants to provide three associates to a given cue (e.g., “cat”). The two additional responses help elicit nondominant associations, with limited evidence of response chaining (i.e., bias from previous responses; De Deyne et al., 2019). SWOW typically presents volunteers with 18 cues that provide responses in the course of minutes. In contrast, we asked each individual participant to provide answers to thousands of cues over the course of weeks. Specifically, each participant in our study was asked to provide three associations to a total of 3,000 unique cues and 600 repeated cues (to assess reliability), resulting in a total of 10,800 responses per participant.

We obtained data for four younger (aged 24 to 28) and four older (aged 68 to 70) native German speakers. In an initial session, participants were briefed in person and received instructions concerning the dedicated online tool that they could use to complete the word associations. Participants then completed the word associations from home over the course of weeks. After finishing the word association task, participants returned to the laboratory for cognitive testing on several memory and linguistic tasks. In particular, we focused on verbal fluency, paired associated learning, and episodic memory tasks that have often been used to estimate and understand individual and age differences in cognitive performance (Ramscar, Sun, Hendrix, & Baayen, 2017; Zemla & Austerweil, 2018). A companion data paper provides a detailed account of our design, participants, and methods (Wulff, Aeschbach, Deyne, & Mata, in press). The data can be downloaded from https://osf.io/vkwps/.

### 4.1 Differences in the semantic networks of younger and older individuals

In what follows, we document a first effort to obtain individual semantic networks from word association data in MySWOW and report a qualitative comparison of the results with those from past work, which suggests structural differences between the semantic representations of younger and older adults (Dubossarsky et al., 2017; Wulff et al., 2019).

**Fig. 2:**
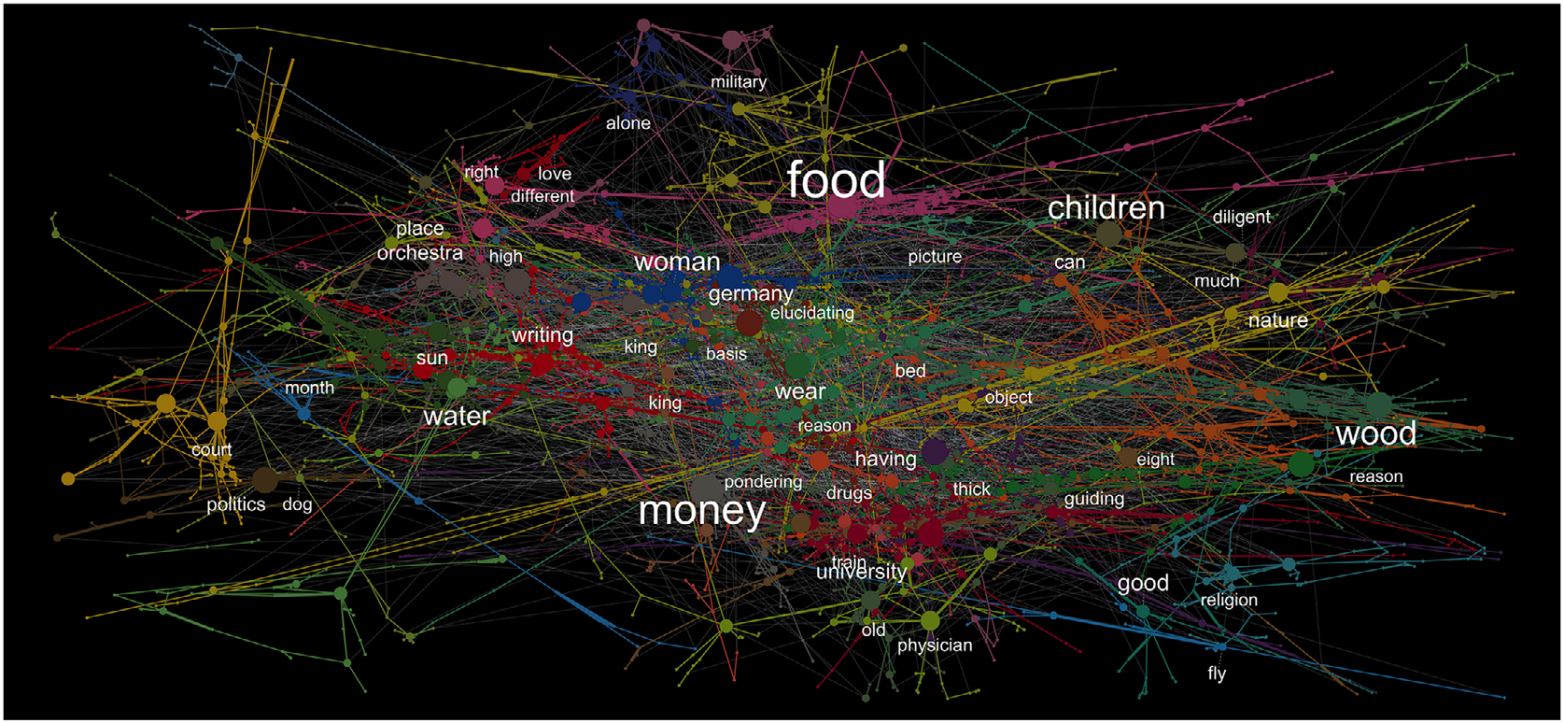
Associative network of one participant. Node size represents PageRank centrality, colors communities detected using the Louvain algorithm (Blondel, Guillaume, Lambiotte, & Lefebvre, 2008). Words were translated from the original German and show the most important words in the respective community, with size representing the word's importance in the overall network as determined by PageRank.

For each individual, we created unipartite networks by placing weighted, undirected edges between responses and corresponding cues. This resulted in eight individual networks containing 4,836 to 6,461 nodes, which is comparable in size to previously obtained large‐scale aggregate associative networks (Morais et al., 2013; Steyvers et al., 2005). Fig. 2 illustrates the network of one individual, highlighting the most central words according to PageRank (e.g., Griffiths, Steyvers, & Firl, 2007) and the underlying structure by identifying clusters extracted using the Louvain method (Blondel et al., 2008).

Networks strongly overlapped in content with each other and with existing SWOW data sets. For instance, the top 10 most central words in the German SWOW data set,^2^ *money*, *music*, *work*, *school*, *water*, *car*, *love*, *green*, and *important*, are all found among the top 100 most central words in all individual networks, while also making up 38% of the top 10 most central words in individual networks. Consistent with previous work (e.g., Dubossarsky et al., 2017; Wulff, Hills, Lachman, & Mata, 2016; Wulff et al., 2018), older adults' networks were larger ($N_{OA}=5,921$ vs. $N_{YA}=5,133$), had lower degrees (${\left\langle k\right\rangle }_{OA}=1.39$ vs. ${\left\langle k\right\rangle }_{YA}=1.66$), had lower clustering coefficients ($C_{OA}=0.053$ vs. $C_{YA}=0.115$), and had larger shortest path lengths ($L_{OA}=8.87$ vs. $L_{YA}=7.45$) than those of younger adults (see Table 2). Except for one older adult, whose network had structural characteristics more similar to those of the younger adults (row 5 in Table 2), these patterns held for all possible pairwise comparisons of younger and older adults' networks. Also consistent with earlier work, the degree distributions^3^ of older adults' networks were less similar to each other (.48<r<.58) than those of younger adults (.58<r<.71), suggesting a progression of network differentiation with age (Wulff et al., 2018).

**Table 2:** Macroscropic differences in semantic network structure in terms of degree (k), clustering coefficient (C), and average path length (L) for full and common individual networks based on nodes shared among participants

		Full network				Common network			
Group	Age	|V|	⟨k⟩	C	L	|V|	⟨k⟩	C	L
Young	24	5,780	3.03	.091	8.15	2,111	3.03	.153	7.66
Young	27	4,836	3.47	.115	7.13	2,111	3.55	.174	6.70
Young	27	4,920	3.31	.119	7.36	2,111	3.32	.202	6.81
Young	28	4,995	3.44	.136	7.14	2,111	3.62	.220	6.54
Old	68	5,275	3.35	.059	6.54	2,111	3.33	.110	5.94
Old	68	6,461	2.63	.045	9.33	2,111	2.46	.072	8.86
Old	69	6,157	2.78	.053	8.37	2,111	2.76	.093	7.76
Old	70	5,792	2.39	.055	11.3	2,111	2.19	.114	10.8

## 5 Linking individual networks to cognitive performance

Can the networks described above be used to understand individual cognitive performance? This question can be addressed at two levels: At the network level, macroscopic properties of the network, such as average degree or clustering, could be used to predict overall performance in a given task (Kenett & Faust, 2019). At the node level, microscopic properties, such as node degree or the shortest path length between two nodes, can be used to predict trial‐level performance, such as the order of retrievals in a verbal fluency task. As the small sample size (N=8) of our proof‐of‐concept study limits comparisons at the network level, we focus on relating semantic networks and cognitive performance on the node level. To this end, we analyze how well two important node characteristics—namely, node centrality, measured using PageRank (see, e.g., Griffiths et al., 2007), and node similarity, measured using Katz' walk similarity (see, e.g., De Deyne, Navarro, Perfors, & Storms, 2016; Hills, Jones, & Todd, 2012)—correspond to response patterns in two verbal fluency tasks, a paired associative learning task and an episodic memory task.

**Fig. 3:**
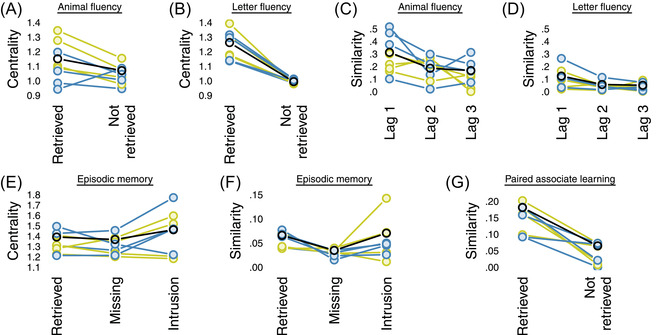
Links between mental representations and cognitive performance for the four younger (blue lines) and the four older adults (yellow lines). The panels show the difference in PageRank centrality between retrieved and nonretrieved words in animal (A) and letter fluency (B), the average cosine similarity of words one, two, and three words apart in animal (C) and letter fluency (D), the PageRank centrality of retrieved, missing, and intrusion words in the episodic memory task (E), the average cosine similarity of retrieved, missing, and intrusion words to (other) retrieved words in the episodic memory task (F), and the average cosine similarity of retrieved versus nonretrieved word pairs in the associative recall task (G). The black line shows the effects under the aggregate representations. Centrality is measured as PageRank times the number of nodes in the network, to account for differences in network sizes.

In the animal and letter verbal fluency tasks, individuals were asked to retrieve as many animal words or words starting with the letter S, respectively, as they could, within 10 min.^4^ The retrieved words had substantially higher centrality than words that were not retrieved, but are in an individual's network. Specifically, for seven out of eight individuals, retrieved animals were more central in their respective networks as compared to other animals contained in the respective networks that were not retrieved by the individual (Fig. 3A). Similarly, for all eight individuals, retrievals of words starting with the letter S had higher centrality in the respective networks than other words starting with any same letter contained in their networks (Fig. 3B). We also found words occurring directly adjacent within retrieval sequences to be more similar to each other than words more distant from each other. Comparing directly adjacent words (Lag 1) to words that were three responses apart, this was the case for every individual in the animal fluency task (Fig. 3C) and for six of the eight individuals in the letter fluency task (Fig. 3D). In the episodic memory task, participants freely recalled words from previously studied word lists.^5^ Both retrieved words and intrusions, that is retrieved words that were not on the list, but included in the individual's network, tended to have, on average, higher centrality than missing words (Fig. 3E). This pattern held for five out of eight individuals. Furthermore, both retrieved words and intrusions were, on average, more similar to other retrieved words than to missing words (Fig. 3F) for seven of eight individuals. Finally, the associate recall task required individuals to retrieve previously learned word pairs.^6^ Retrieved word pairs had, on average and for all eight individuals, higher similarity than words pairs that were not retrieved (Fig. 3G).

Each of these links demonstrates that individual networks can be used to predict an individual's pattern of behavior in several cognitive measures. The critical question arising from this framework, however, is whether individual networks reveal idiosyncratic differences that can be used to understand an individual's behavior better than would be possible using an aggregate representation derived from the responses of many individuals. To assess this, we compared the magnitude of the effects presented in Fig. 3, for example, the PageRank of retrieved versus nonretrieved words in verbal fluency tasks, which were derived using individuals' personal networks, against the magnitude of effects resulting from using an aggregate derived from combining the responses from all eight individuals. This analysis revealed that the aggregate network produced, on average, equally large, if not larger, effects than individual networks (see black lines in Fig. 3).

The similar or improved performance of the aggregate compared to individuals' own networks could be due to differences in both the reliability and precision of measurement of the different types of networks. First, on average, only 53% of three possible associations given to repeated cues were also given during the first encounter of the same cue, indicating, at best, moderate levels of internal consistency. Furthermore, focusing on the 2,111 shared nodes, we found individuals' networks to share a large amount of variance with the aggregate network, as indicated by average correlations of $\bar{r}=.61$ and $\bar{r}=.55$ for PageRank and cosine distributions, respectively, although variance shared with the networks of other individuals was, on average, rather low (PageRank: $\bar{r}=.34$; Cosine: $\bar{r}=.33$). These results suggest that the individual networks contain a shared component that is reliably captured by an aggregate network. They also suggest that the aggregate network is relatively free from aggregation bias (see also Wulff et al., 2018). Second, the aggregate network derived from the response of all individuals is given access to more detailed information on the relative strength of associations between nodes in the networks. For individuals, only 2.2% to 5.5% of word associations occurred more than once, and these repetitions occurred a maximum of two or three times per individual. For the aggregate representation, however, 20.5% of associations occurred more than once, with counts reaching as high as 15, leading to a more graded pattern of edge strengths between network nodes in the aggregate relative to the individual networks. One should note that this implies that the comparison of individual and aggregate networks is largely confounded with a comparison of weighted (aggregate) and unweighted (individual) networks.

## 6 What have we learned from MySWOW so far?

Our approach using network science was successful in mapping large‐scale individual networks for a small sample of younger and older adults, suggesting that the approach can be used to capture the mental representations of individuals: Individuals' networks were highly similar to each other in terms of content, and these contents were qualitatively similar to those of past efforts to produce aggregate networks, such as SWOW. In line with past work, the results from the eight participants suggested age‐related differences in their structural composition (Dubossarsky et al., 2017; Wulff et al., 2018). All in all, our approach seems promising in offering a window into adult age differences in the content and structure of the aging lexicon and the individual differences that can arise across adulthood as a function of cumulative experience.

Our results also showed that estimates of node centrality and relatedness derived from single individuals' networks exhibited strong links to individuals' responses in four cognitive tasks, including two verbal fluency and two memory tasks. These results demonstrate that it is feasible, in principle, to map information from individual semantic networks onto additional linguistic and memory performance at the single participant level. Naturally, future work needs to strive to collect data from more individuals to assess whether network‐level properties are systematically related to differences in cognitive performance *between* individuals.

Our results demonstrate that our approach is, nevertheless, limited. Most crucially, our results suggest that individual networks were less powerful in accounting for individuals' response patterns in the additional measures of linguistic and memory performance than an aggregate network composed of all individuals' responses. The advantage of aggregate over individual networks presents a major challenge to the claim that it is important to assess individual networks to understand individual differences in cognitive performance. Before one fully dismisses our claim, one should distinguish two explanations for these findings that have different implications. First, one possibility is that our approach was not able to capture relevant individual differences in the content of the semantic representations, for example, because of the use of a pool of cues containing mostly high‐frequency words for which individual differences in semantic representations may be small. In this case, the breadth of cues used would need to be expanded so that the networks generated are better able to capture the idiosyncratic nature of individuals' representations. Second, another possibility is that our approach may not have been able to measure the strength of association between various nodes (i.e., edges) with sufficient precision and reliability. In this case, additional work may be needed that considers both obtaining larger sets of responses per cue, having participants provide responses to the same cue on repeated occasions, or asking individuals to provide additional ratings (Roads & Love, 2020). In our view, these possibilities speak to the need for future work that guarantees appropriate scope and reliability rather than a clear rejection of the idea that individual networks can be helpful to understanding the link between experience and cognitive performance.

## 7 General discussion

We proposed an empirical agenda that utilizes network science to provide an estimate of the extent to which idiosyncratic experiences matter for the size and structure of mental representations and, ultimately, individual and age differences in cognitive performance. Despite the promise we see in this approach, and some encouraging results from our feasibility study, a number of major challenges remain.

First, there are still considerable questions about how to best capture individual semantic networks. As discussed in Section 1, the MySWOW approach is only one of several: There are alternative elicitation methods (see Table 1) as well as different network estimation methods (e.g., Zemla & Austerweil, 2018) that merit further consideration. Future work may want to rely on several of the available methods and compare commonalities and differences of their results to obtain a better picture of individual networks. One limitation that may be common to many of the currently available approaches, however, is that no elicitation of individuals' knowledge store is independent of the process by which the representation is accessed. As a result, any individual or age differences detected cannot be unequivocally assigned to the nature of association in the knowledge store (representation), but can in principle result from the process by which this representation is searched and accessed (Jones, Hills, & Todd, 2015; Kenett, Beckage, Siew, & Wulff, 2020; Kraemer, Wulff, & Gluth, 2021; Siew, Wulff, Beckage, & Kenett, 2019). So far, there seems to be no clear consensus concerning the extent to which representation and process are entangled in the kinds of tasks typically used to elicit semantic networks (Abbott, Austerweil, & Griffiths, 2015; Jones et al., 2015). The degree of entanglement likely depends on the nature of the tasks and processes compared. At least in word association tasks, empirical evidence suggests associative strength between word pairs in itself is a poor predictor of a wide variety of findings that involve retrieval about these pairs from semantic memory (De Deyne et al., 2019). The reason for this is that the same retrieval processes underestimate semantic effects due to frequency biases (i.e., a frequent word like money is the response to many cue words, and therefore carries little information). In most studies, such biases need to be mitigated using appropriate transformations of associative strength that highlight cue‐specific information (e.g., point‐wise mutual information; De Deyne et al., 2019). More generally, it is unlikely that a single approach can provide a definitive resolution to this conundrum and there are two possible ways forward that we would like to emphasize. One potential way to distinguish the role of structure versus process could be to use multiple methods of assessment (e.g., word association, verbal fluency, relatedness judgments) to elicit mental representations. To the extent that these different assessment methods are associated with different search processes and strategies, convergent evidence could provide some support for the role of representation. A related approach could involve the use of the same elicitation method under different explicitly instructed search strategies that could equate these across participants (Wulff, Hills, & Hertwig, 2013, 2020). A second direction could involve using techniques from neuroscience to distinguish between search (i.e., control) processes and representational components (Hoffman & Morcom, 2018), in particular to the extent that individuals' representational space has a signature in the functional organization of the brain (Huth, Nishimoto, Vu, & Gallant, 2012).

Second, we are still lacking a computational model of learning and cognition that captures and links all aspects of interest (i.e., environment–representation–performance). A suite of models would be ideal because they could be used to generate predictions about which tests should be used to assess individual and age differences in mental representations as a function of experience. These predictions could then guide specific empirical approaches and tests. These models may also be generative in allowing us to test specific components contributing to individual differences, such as the role of learning, memory, or search strategies (Wulff et al., 2019). For example, past work has used techniques from network science that assess the robustness of networks to perturbation and decay that could be instrumental in understanding the role of aging processes, such as forgetting (Borge‐Holthoefer, Moreno, & Arenas, 2011; Cosgrove et al., 2021).

Third, there is still a dearth of data concerning single individuals' exposure to the physical, linguistic, and social environment that can then be linked to individuals' mental representations. Most past work has used simple measures of self‐reported exposure (e.g., to print; Mol & Bus, 2011) but there may be more efficient alternatives that are now available, for example, experience sampling (Dennis, Yim, Garrett, Sreekumar, & Stone, 2019), or massive recording and analysis of naturalistic linguistic exposure, such as speech (Mehl, 2017) and text (Banda et al., 2021). These approaches could become particularly powerful as such data become increasingly connected with other information about individuals, for example, if these are collected with informed consent in larger surveys or longitudinal household panels that also include dedicated cognitive instruments, such as verbal fluency or memory tasks (Taler, Johns, & Jones, 2020).

Fourth, and finally, most of past research and our own approach introduced above is correlational in nature in that it aims to establish a correlation between individual experience and the contents and structure of mental representation or cognitive performance. In order to help establish causality, however, one would need a comparison of individuals assigned to different environmental exposures through either natural or, ideally, controlled experiments. There are a few candidate training strategies, including training of specific physical or virtual environments (Miller et al., 2013), artificial or natural languages (Pothos, 2007), and even complex narratives (Heusser, Fitzpatrick, & Manning, 2021). Regardless of the exact type of information and mode of exposure, it remains a challenge to obtain meaningful and reliable individual estimates of the emergent mental representations and their potential effects across large swathes of time such as a life span.

## 8 Conclusion

We have argued that quantifying individual and age differences in the size and structure of human knowledge is important because this represents a missing link in estimating the role of cumulative experience in cognitive performance. We specifically proposed an empirical agenda that combines tracking individuals' idiosyncratic experiences, broad mapping of their mental representations using the tools of network science, and studies linking representational structure to individuals' cognitive performance. We strongly believe such steps can move us closer to understanding the role of the environment in shaping the structure of the mental lexicon and its implications for cognitive aging.

### Open Research Badges

This article has earned Open Data. Data is available at https://osf.io/vkwps/.

## Notes

1 A recent alternative approach (Roads & Love, 2020) that relies on ranking and active learning algorithms can provide a solution to the problem of scale.

2 German SWOW data were downloaded on January 25, 2021.

3 Considering only the 2,111 words shared between all individual networks.

4 On average, between 62 and 113 animals and between 45 and 138 words starting with the letter S were retrieved. Animals overlapped with 1.5% of cues and 0.8% of responses, whereas words starting with the letter S overlapped with 11.1% of cues and 11.9% of responses. For details see Wulff et al. (in press).

5 Participants correctly recalled between 28.7% and 60.9% of words, with an additional 1.3% to 25% intrusions. For details see Wulff et al. (in press).

6 Participants correctly recalled between 32.8% and 96.8% of pairs. For details see Wulff et al. (in press).
